# Time pressure effects on decision-making in intertemporal loss scenarios: an eye-tracking study

**DOI:** 10.3389/fpsyg.2024.1451674

**Published:** 2024-10-11

**Authors:** Yan-Bang Zhou, Shun-Jie Ruan, Kun Zhang, Qing Bao, Hong-Zhi Liu

**Affiliations:** ^1^Institute of Teacher Education, Ningxia University, Yinchuan, China; ^2^School of Journalism and Communication, Ningxia University, Yinchuan, China; ^3^Laboratory of Behavioral Economics and Policy Simulation, Nankai University, Tianjin, China; ^4^Department of Social Psychology, School of Sociology, Nankai University, Tianjin, China

**Keywords:** decision-making, time pressure, eye-tracking, loss, intertemporal decision making

## Abstract

This study utilized eye-tracking techniques to investigate decision-making behavior in intertemporal loss scenarios under both time pressure and no time pressure conditions. Results revealed shorter decision-making times and decreased large later (LL) option selection frequency under time pressure. Participants under time pressure exhibited reduced Mean Fixation Duration (MFD) and Search Measure (SM) values, indicating altered information processing. Mediation analyses confirmed that task choice outcomes were influenced by SM and MFD, suggesting a shift towards heuristic decision-making under time pressure.

## 1 Introduction

Ants need to store food to survive the future winter, while humans need to make decisions that will impact the future (McClure et al., 2004; Mannetti et al., 2009; Dholakia et al., 2016). When making decisions, humans must consider constraints such as time, knowledge, and cognitive abilities. However, many important decisions are influenced by time constraints, requiring decision-makers to make intertemporal choices quickly (Svenson et al., 1990). Intertemporal choice is an important and pervasive concept that refers to decisions involving tradeoffs between different time costs and benefits (Frederick et al., 2002). Time pressure, as a form of stress, influences individuals’ cognition during decision-making, leading to differences in decision outcomes. For instance, in the domain of intertemporal decision-making, individuals under time pressure exhibit more patience (Lindner and Rose, 2017). In the domain of risky decision-making, individuals under time pressure exhibit more risk-seeking (Young et al., 2012; Saqib and Chan, 2015; Madan et al., 2015; Lin and Jia, 2023). The main reasons may include the following aspects. Firstly, time constraints directly impact neural activity in cognitive regions (Jocham et al., 2014). Additionally, individuals adopt corresponding strategies to cope with this pressure, such as employing heuristic strategies (Verplanken, 1993; Bobadilla-Suarez and Love, 2018). For instance, in intertemporal gain decisions, individuals under time pressure tend to favor heuristic strategies, choosing larger future gains (Lindner and Rose, 2017). In the area of risky decision-making, individuals more frequently choose the sure option for gains and the gamble option for losses when there is greater pressure to make quick decisions, this framing effect is driven by the heuristic strategy (Guo et al., 2017). Furthermore, some scholars have provided related explanations from the perspective of attention. Individuals under time pressure are more attentive to negative information on important dimensions compared to those without time pressure (Svenson and Edland, 1987). They also tend to adopt non-compensatory strategies to adapt to time pressure (Svenson et al., 1990; Pieters and Warlop, 1999).

Loss and gain are fundamentally different, with distinct decision mechanisms. When individuals face intertemporal decisions involving loss or gain, there are significant differences in brain activity (Xu et al., 2009). As described by the framing effect, the framing of the same event in different ways can lead to vastly different decision outcomes. For example, when framed as losing $100 in 30 days versus gaining $100 in 30 days, individuals exhibit different discount rates (Tversky and Kahneman, 1981; Pachur and Kellen, 2013). Currently, researchers have primarily focused on intertemporal decision-making under time pressure in the domain of gains. However, the mechanism by which time pressure affects intertemporal decision-making in loss scenarios remains unclear. When individuals make choices between economic, environmental, and health gains and losses, the discount rates for gains are higher than for losses (Hardisty and Weber, 2009). Additionally, compared to immediate gains, people perceive delayed losses with lower intensity (Hardisty et al., 2013). Due to aversion to loss, individuals not only experience stronger negative emotions when making loss decisions, but also tend to terminate losses more quickly in lower discount rates (Zhao et al., 2018). Thus, the framing differences between losses and gains lead to asymmetries in behavioral outcomes. In real life, people often encounter situations involving losses, such as deciding whether to handle a traffic violation promptly or postpone it, or scheduling surgeries.

Time pressure objectively limits the time for deliberation, thereby making decisions more difficult. Just as depicted in Rodin’s sculpture “The Thinker,” there are situations where we need to make effort to find solutions to problems. While sometimes, using our intuition and experience, we were able to make decisions quickly and efficiently. The former represents a slower, more effortful decision system, while the latter represents a quick and effortless decision system (Frankish and Evans, 2009; De Neys and Pennycook, 2019). Dual-systems theory propose present-biased preferences to evolve from the interplay of an affective and a deliberate system, where the latter can be disrupted in its operations by time pressure (Kahneman and Frederick, 2002). In other words, individuals experiencing time pressure tend to adopt intuitive, rapid decision-making strategies that facilitate more effective decision-making (Finucane et al., 2000). Dual-system theory provides the most direct theoretical basis for explaining decision-making under time pressure (Guo et al., 2017; Teoh and Hutcherson, 2022). Some researchers use dual-system theory to explain the framing effect under time pressure, suggesting that individuals under time pressure adopt intuition-based decision-making strategies and exhibit a stronger framing effect (Guo et al., 2017; Roberts et al., 2021). For the study of dual systems, the eye-tracking experimental method is a desirable approach. Eye-tracking technology has clear advantages in studying dual-systems by comparing eye movement differences between two decision modes (Horstmann et al., 2009). This comparison is also applicable in the medical field (Logiudice et al., 2021) and consume decision-making under time pressure (Pieters and Warlop, 1999). Thus, the use of eye-tracking technology can deeply explore the differences in dual-system decision processes under time pressure.

In summary, we plan to investigate the attentional mechanisms of intertemporal decision-making under time pressure using eye-tracking technology. Firstly, individuals face loss situations more frequently in real-life. Secondly, based on existing research, individuals tend to exhibit more patience in intertemporal decision-making regarding gains under time pressure (Guo et al., 2017; Lindner and Rose, 2017), attributed to the asymmetry between losses and gains (Tversky and Kahneman, 1981). Intertemporal loss decisions under time pressure may therefore lean towards faster loss acceptance, but this remains insufficiently explored. Lastly, the mechanism of intertemporal loss under time pressure remains unclear, with eye-tracking technology being relatively suitable. For these reasons, we intend to utilize eye-tracking technology to explore the impact of time pressure on intertemporal decision-making within the loss framework. Specifically, whether time pressure leads to decision reversals and the underlying attentional mechanisms will be investigated. Under the gain framework, individuals are more cautious under time pressure compared to situations without time pressure, and they tend to choose the large later (LL) option (Guo et al., 2017; Lindner and Rose, 2017). Therefore, we hypothesize that in the loss framework, the choice of the LL option is significantly lower under conditions with time pressure than under conditions without time pressure.

Furthermore, to examine the differences in attention mechanisms under conditions with and without time pressure, we employed two eye-tracking indexes. The first index, Mean Fixation Duration (MFD), reflects the average duration of a single fixation during a decision-making process, used to describe the depth of information processing during decision-making (Amblee et al., 2017; Zhou et al., 2021, 2022). Typically, heuristic strategies are associated with shorter MFD, while deliberative strategies are associated with longer MFD (Horstmann et al., 2009; Su et al., 2013). The second index is the Search Measure (SM) Index, used to measure whether information search direction in a decision-making process is based on dimensions or options (Liu et al., 2021). Option-based processing, which focuses on overall assessment, and dimension-based processing, which focuses on attribute comparison, represent two different approaches to information processing and decision-making. A higher SM value indicates a decision based more on options, while a lower SM value indicates a decision based more on dimensions. SM values follow a standard normal distribution and have been extensively utilized in eye movement studies related to decision-making processes (Su et al., 2013; Konstantinidis et al., 2017; Schulte-Mecklenbeck et al., 2017). Besides, SM value is more sensitive to changes in search patterns than other metrics. Indeed, if the search strategy is manipulated so that the dominant search pattern switches, the SM value is effective in detecting such changes (Böckenholt and Hynan, 1994). Due to the influence of time pressure, individuals tend to employ dimension-based non-compensatory strategies for quick decision-making to adapt to decision requirements (Svenson et al., 1990). Therefore, heuristic strategies may be associated with smaller SM values, while deliberative strategies may be associated with larger SM values. Under the time pressure, individuals may exhibit differences in both information processing depth and search direction. Hence, we propose hypothesis 2: under the loss frame, MFD is significantly higher without time pressure compared to with time pressure. Similarly, we propose hypothesis 3: under the loss frame, SM values are significantly higher without time pressure compared to with time pressure. To further explore the impact process of time pressure on decision outcomes, we predict behavioral outcomes using eye-tracking metrics (Sui et al., 2020; Zhou et al., 2021; Wei et al., 2022). Time pressure influences the proportion of selecting the LL option through MFD and SM, thus we propose hypothesis 4: the proportion of selecting the LL option is mediated by MFD and SM under conditions with and without time pressure.

## 2 Methods

### 2.1 Participant

The necessary sample size was determined using G*Power software (Faul et al., 2007) through linear regression analysis, assuming a medium effect size (Cohen’s f^2^ = 0.15), which indicates a moderate level of practical significance. The analysis indicated that 55 participants were required to achieve a power of 0.80 at a significance level of 0.05. To ensure robustness and account for potential attrition, 72 participants were recruited, including 28 males, with an average age of 20.9 years. Participants were given 10,000 units of virtual currency (approximately US$10) as a decision-making reward. At the end of the experiment, two trials were randomly selected from their real choices, and deductions based on their performance were applied. On average, participants retained 8,000 units (approximately US$8). The study received approval from the Ethics Committee, and all participants provided signed informed consent before participating.

### 2.2 Apparatus

During the experiment, eye movement data from participants were captured using an EyeLink1000 Plus eye tracker (SR Research, Ontario, Canada). The stimuli were displayed on a 20-inch Dell monitor with a resolution of 1,280 × 1,024 pixels and a refresh rate of 60 Hz, offering a viewing angle of 36° horizontally and 29° vertically. Participants were seated approximately 58 cm from the screen and were instructed to minimize head movements by utilizing a chin rest. Although participants observed the stimuli with both eyes, data collection was restricted to the right eye only. Responses to the stimuli were recorded through keyboard input.

### 2.3 Stimuli

The experiment employed a computer to generate 10,000 intertemporal options. Each option comprises a delay and an outcome, representing a specific financial loss incurred at a designated point in time. These options were then randomly paired into sets of small sooner (SS) and large later (LL) options. Following the removal of strongly dominant option pairs, 50 pairs of SS and LL options were randomly chosen from the remaining pairs. The options were presented using Arial font, ensuring a viewing angle of 2.89° and a minimum distance of 5° between any two stimulus presentations (Rayner, 1998, 2009). Additionally, to encourage conscientious participation, five pairs of strongly dominant options were included in the task. To ensure balanced presentation of the experimental material, half of the participants viewed the delayed option above the outcome, while the remaining half observed the outcome positioned above the delay.

### 2.4 Task

We employed a within-participant design, with time pressure as the independent variable and choice outcomes and eye-tracking measures as the dependent variables. Participants were tasked with selecting their preferred option between SS and LL options. Each participant completed both choice tasks, one under no time pressure and the other under time pressure. Half of the participants commenced with the no time pressure task, while the remaining participants initiated with the time pressure task. In the time pressure task, participants were required to make decision within 3 s of the stimulus presentation (Svenson and Edland, 1987; Madan et al., 2015), while in the no time pressure task, participants had unlimited time to decide. At the conclusion of the experiment, participants indicated their levels of stress on a scale ranging from 1 (not enough time) to 9 (more than enough time) (Saqib and Chan, 2015).

To incentivize full engagement with the experiment, participants were informed at the beginning that they would receive 10,000 units of virtual currency for decision-making. Upon completion of the experiment, two trials were randomly selected. Depending on the participant’s choice in the selected trial, the delay and outcome were discounted as the actual loss (Zhou et al., 2021, 2022).

### 2.5 Experimental procedures

Initially, participants were directed to the laboratory and instructed to read and sign an informed consent form. After this, experimental instructions were displayed on the laboratory computer screen for the participants to review. An experimenter then assisted participants in adjusting their seat height and fixing their head position to ensure comfort. Subsequently, a 5-point calibration was performed to achieve eye fixation, followed by a practice session to familiarize participants with the experimental tasks.

Both the time pressure and no time pressure formal experiments comprised two blocks of 55 trials each. Participants completed the blocks in a random order, with a brief two-minute interval in between.

**Figure 1:**
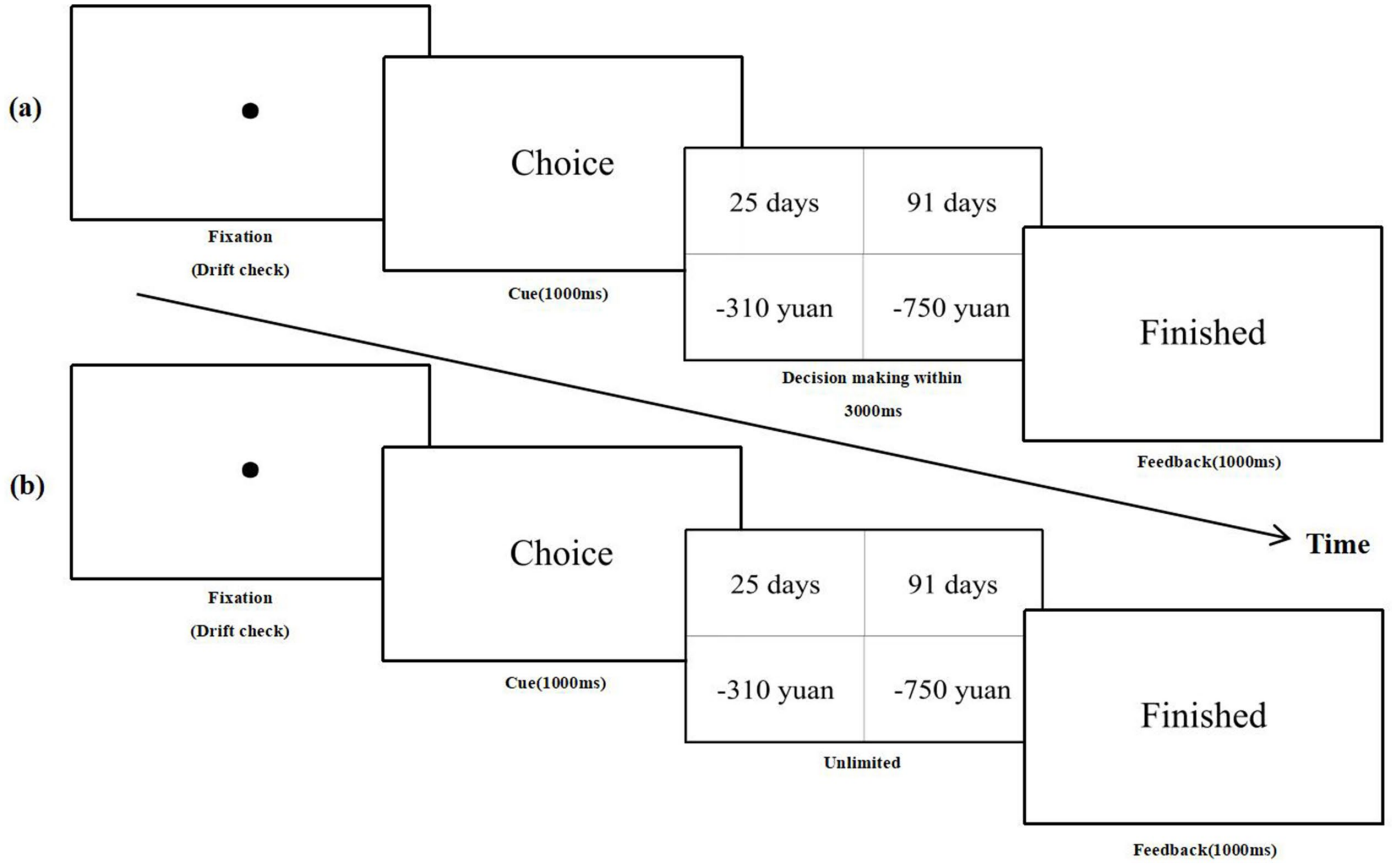
**(a)** A trial flow under time pressure; **(b)** a trial flow under no time pressure.

During the no time pressure task, a fixation point appeared in the center of the screen, prompting participants to fixate and press the space bar. Following this, the word “Choice” was displayed at the center of the screen for 1,000 milliseconds, allowing participants unlimited time to make their selection. Two options then appeared on the left and right sides of the screen, with the F key used to select the left option and the J key for the right option. After a key press, the term “Finish” appeared in the center of the screen for 1,000 ms, signifying the end of the trial (see Figure 1b).

During the time pressure task, participants fixated on a focal point displayed at the center of the screen and pressed the space bar key. This action triggered the appearance of the word “Choice” for 1 s. Following this, participants were given 3,000 ms to browse and press a key to select one of the options. Failure to respond within the time limit resulted in the word “Warning” being displayed at the center of the screen for 1,000 ms, indicating trial failure. Conversely, if a key was pressed, the word “Finished” appeared for 1,000 ms, indicating the end of the trial (see Figure 1a).

## 3 Results

Participants reported their stress levels on a scale from 1 (not enough time) to 9 (more than enough time) (Saqib and Chan, 2015), with an average stress level of 4. Twenty-five trials were disregarded from the analysis because participants failed to respond within the 3,000 ms limit. Additionally, data from three participants were omitted due to incorrect responses in dominant pairings. The remaining dataset has been shared on the Open Science Framework.^1^

### 3.1 Response time

**Figure 2:**
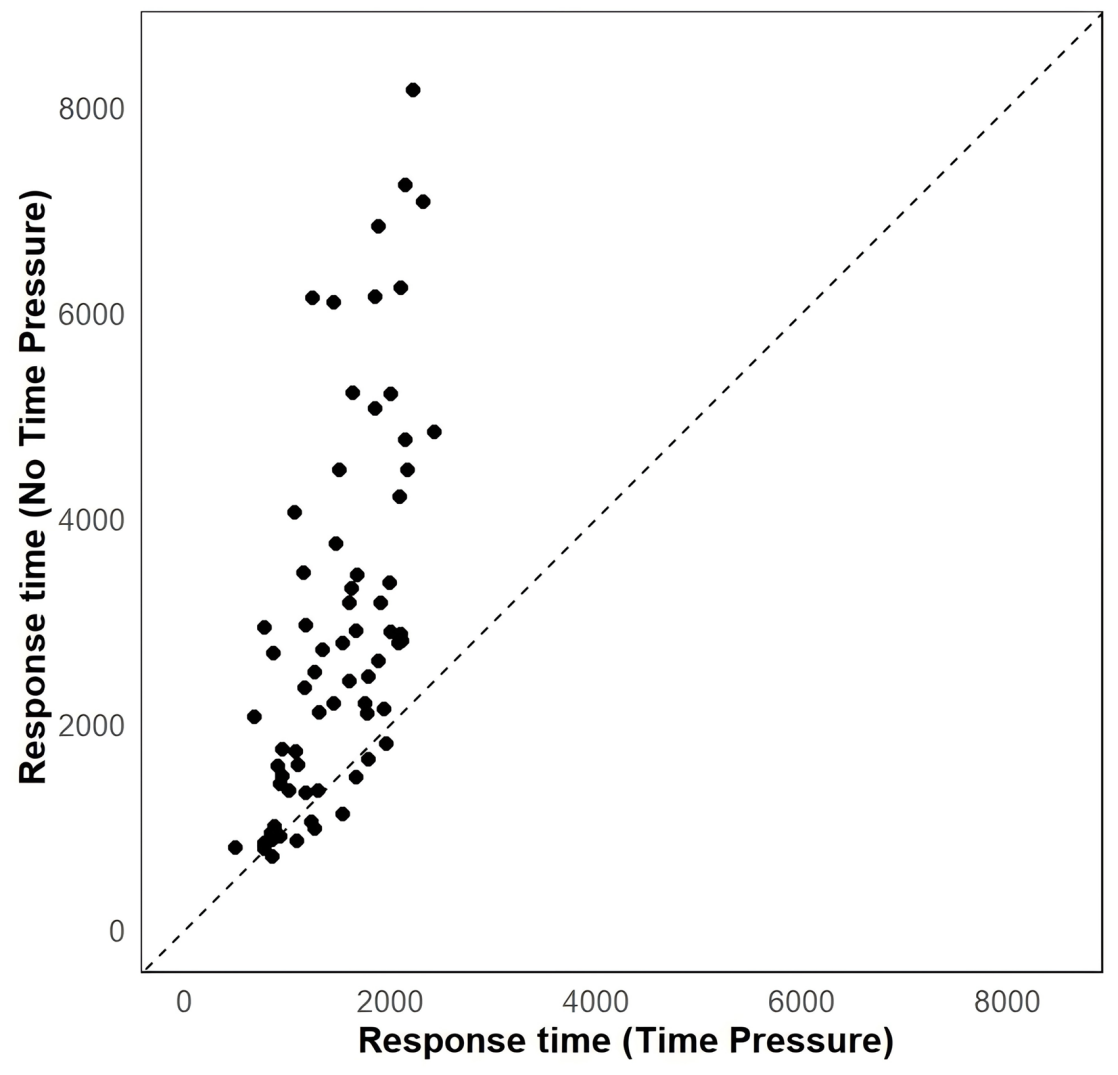
Scatterplots showing the effect of task on response time.

Response time was defined as the duration between the presentation of the option and the key press response. A linear mixed-effects model was used to analyze participants’ reaction times across task conditions. The model included fixed effects for target attributes (1 = time pressure task; 0 = no time pressure task), random effects for participants and items, and used log-transformed reaction times (log (RT)) as the dependent variable. Analyses were performed by using the lme4 and lmerTest packages in the R statistical environment (Kuznetsova et al., 2017). The results revealed that participants spent significantly longer in the no time pressure choice task compared to the time pressure choice task (*b* = −0.489, CI_95%_ = [−0.504, −0.473], M_no time pressure_ = 2,941 ms, M_time pressure_ = 1,480 ms*, t* = −51.76, *p* < 0.001) (see Figure 2). The results indicate that our manipulation of time pressure was effective.

### 3.2 Choice

**Figure 3:**
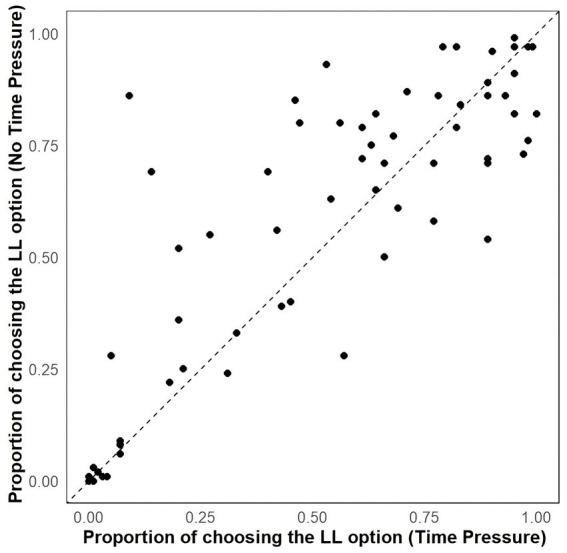
Scatterplots showing the effect of task on proportion of choosing the LL option.

To examine the effect of time pressure on participant choice and to control for individual differences and the number of items, we used a generalized linear mixed model with a binomial family for our analyses. The model included fixed effects for target attributes (1 = time pressure task; 0 = no time pressure task), random effects for participants and items, and used target options as the dependent variable (1 = choose the LL option; 0 = choose the SS option). The results revealed that participants prefer LL option significantly in the no time pressure choice task compared to the time pressure choice task (*b* = −0.228, CI_95%_ = [−0.308, −0.149], M_no time pressure_ = 0.56, M_time pressure_ = 0.51, *z* = −4.749, *p* < 0.001) (see Figure 3).

### 3.3 MFD

**Figure 4:**
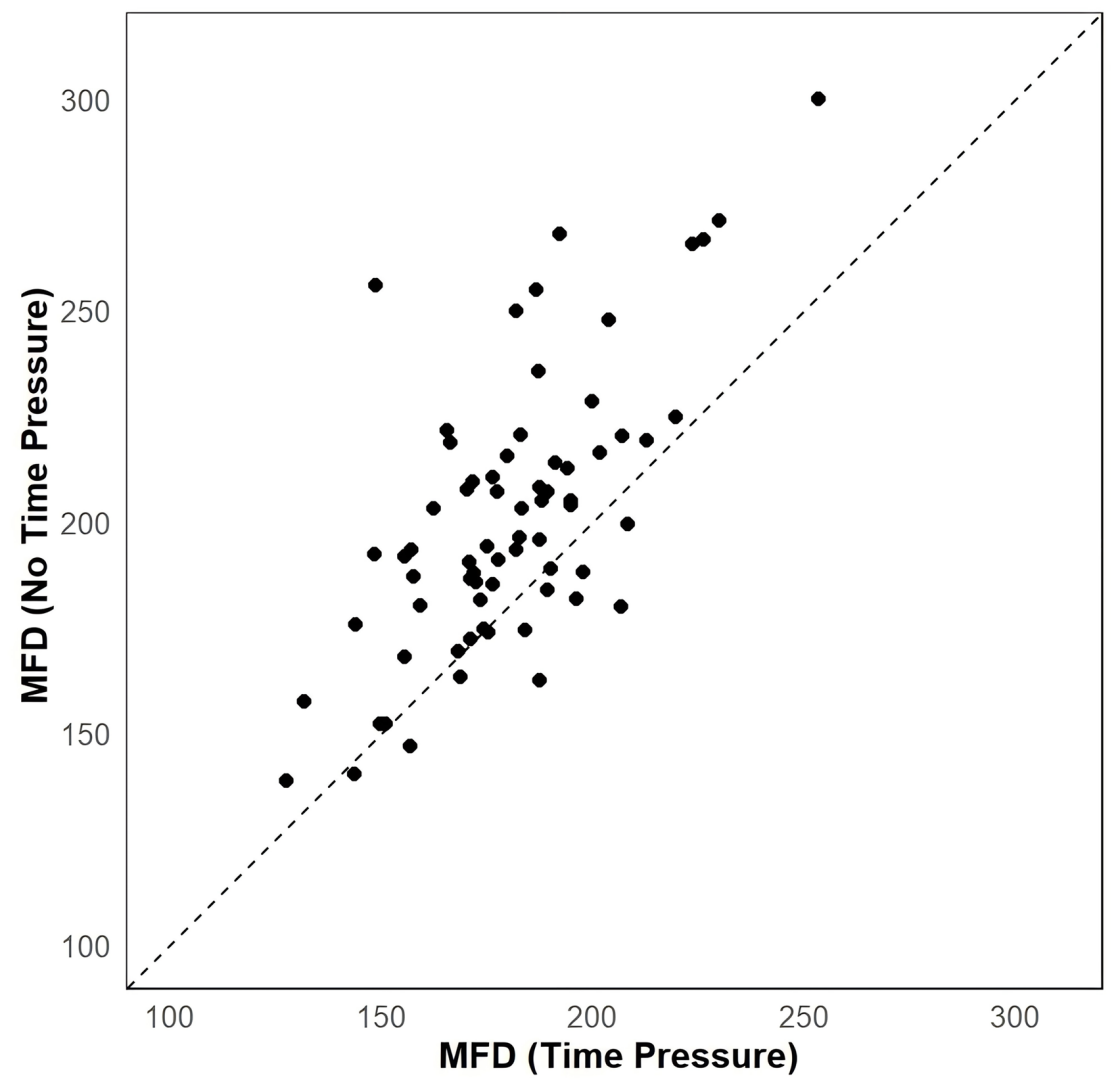
Scatterplots showing the effect of task on MFD.

To assess the level of complexity and cognitive effort involved in information processing across different tasks, we introduced the MFD index variable. MFD represents the mean fixation duration, where a larger MFD indicates greater complexity in participants’ information processing. We examined differences in participants’ Mean Fixation Duration (MFD) values between the two task conditions using a generalized linear mixed model (GLMM) with a Gamma distribution and log link function. The model included fixed effects for task type (1 = time pressure task; 0 = no time pressure task) and random effects for participant and item. The analysis revealed a significant difference in participants’ MFD, with higher values observed in the no time pressure condition compared to the time pressure condition (*b* = −0.106, CI_95%_ = [−0.130, −0.082], *z* = −9.06, *p* < 0.001, M_no time pressure_ = 201.15, M_time pressure_ = 180.20) (see Figure 4). To ensure the appropriateness of our GLMM model, we tested the residuals for normality. The Shapiro–Wilk test (W = 0.992, *p* = 0.663) and the Kolmogorov–Smirnov test (D = 0.051, *p* = 0.872) indicated that the residuals did not significantly deviate from a normal distribution.

### 3.4 SM

To investigate differences in participants’ information search strategies during the decision-making process, we introduced the index variable SM. This index allows comparison of whether participants rely more on dimensions or options when making comparisons. The formula for SM is as follows:


$$SM=\frac{\sqrt{N}\left[\left(\frac{\mathrm{AD}}{N}\right)\left(r_{a}-r_{d}\right)-\left(D-A\right)\right]}{\sqrt{A^{2}\left(D-1\right)+D^{2}\left(A-1\right)}}$$

*A* and *D* in the formula represent the number of options and dimensions, respectively (*A* = 2, *D* = 2). $r_{a}$ represents the frequency of option-based eye-saccades, $r_{d}$ represents the frequency of dimension-based eye-saccades, and N represents the total eye-saccades. A negative value of *SM* indicates that participants preferred an information search pattern based on dimensional comparison, while a positive value indicates that participants preferred an information search pattern based on option comparison (Pachur et al., 2013).

**Figure 5:**
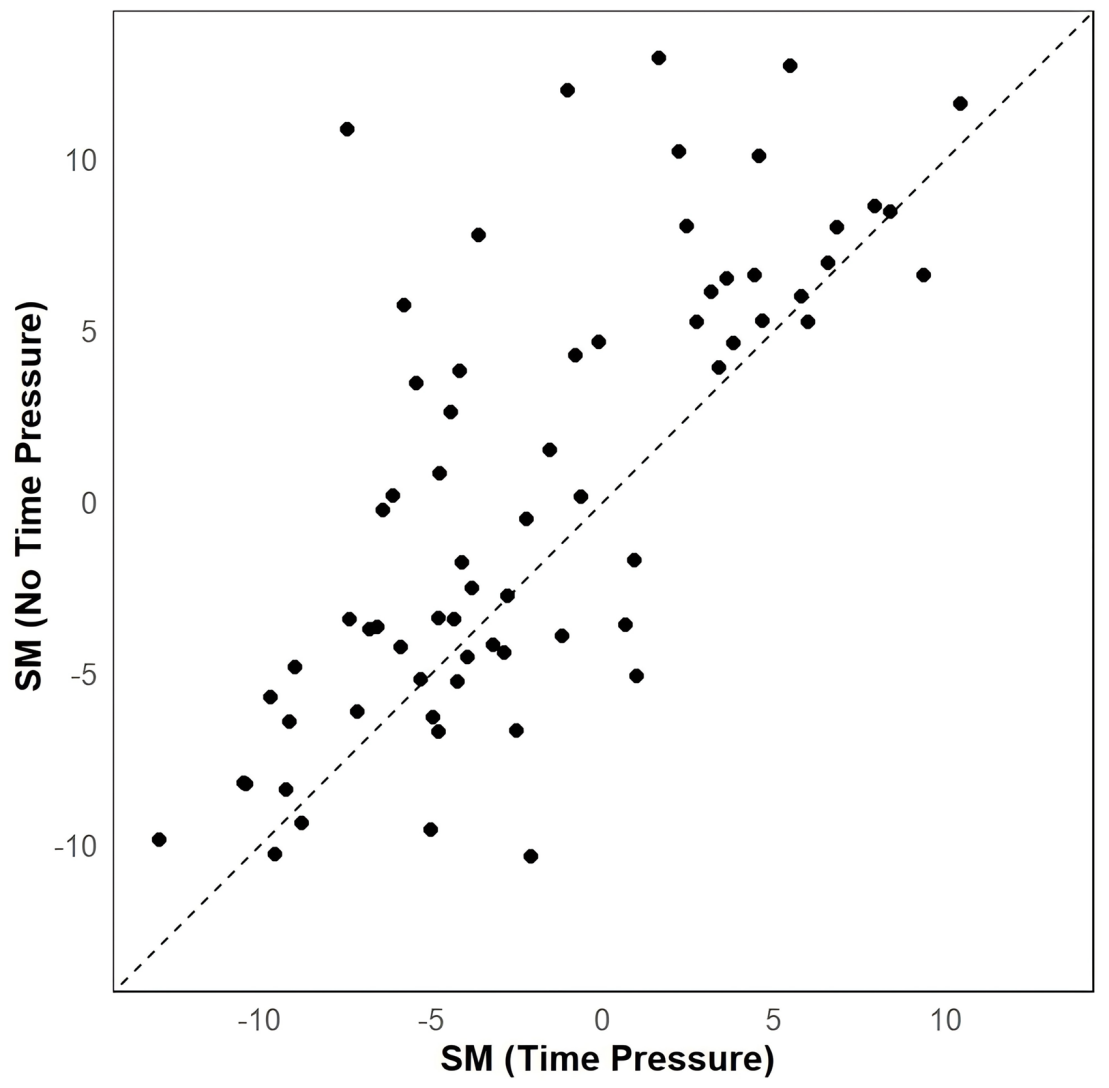
Scatterplots showing the effect of task on SM.

Similarly, we analyzed participants’ SM values between the two task conditions using a linear mixed model. This analysis included fixed effects for task type (1 = time pressure task; 0 = no time pressure task) and random effects for participant number. To address the issue of non-normality in residuals, we applied a square root transformation to the SM values. The analysis revealed a significant difference in participants’ SM values, with higher values observed in the no time pressure condition compared to the time pressure condition (*b* = −0.34, CI_95%_ = [−0.49, −0.18], *t* = −4.22, M_no time pressure_ = 0.29, M_time pressure_ = −1.76, *p* < 0.001) (see Figure 5). Furthermore, the residuals of the model were checked for normality using the Shapiro–Wilk test, which confirmed that the residuals were normally distributed (W = 0.99182, *p* = 0.607). This indicates that participants’ SM values were significantly lower under time pressure compared to no time pressure.

### 3.5 Mediating effect

To investigate whether task type influences the proportion of choosing LL through MFD and SM, we conducted a mediation analysis using M-plus software. We designated the independent variable as task type (1 = time pressure task; 0 = no time pressure task), the mediating variables as MFD and SM, and the dependent variable as the proportion of choosing LL.

**Figure 6:**
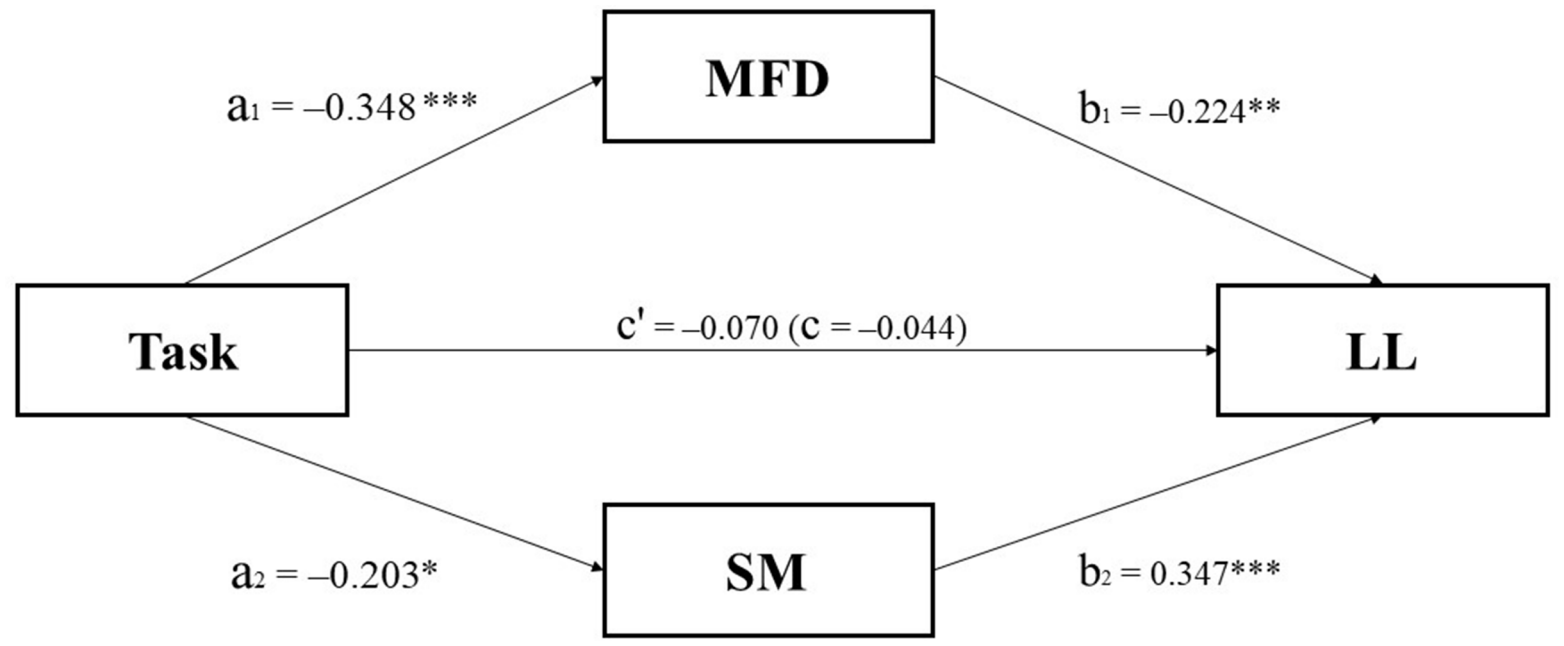
Results of the parallel multiple mediator model analysis of the MFD and the SM. The coefficients in parentheses are the total effect. *p < 0.05, **p < 0.01, ***p < 0.001.

The results revealed significant indirect effects of task on proportion of choosing LL option through the MFD (*a*_1_*b*_1_ = 0.055, CI_95%_ = [0.017, 0.114], *SE* = 0.024, *p* < 0.05) and the SM (*a*_2_*b*_2_ = −0.050, CI_95%_ = [−0.105, −0.011], *SE* = 0.024, *p* < 0.05). The total effect of task on proportion of choosing LL option was not significant (*c* = −0.044, CI_95%_ = [−0.164, 0.073], *SE* = 0.059, *p* > 0.05). The direct effect of task on proportion of choosing LL option (controlling for the influence of the mediators) was not significant (*c’* = −0.070, CI_95%_ = [−0.247, 0.094], *SE* = 0.087, *p* > 0.05) (see Figure 6).

## 4 Discussion

Our study used eye-tracking techniques to investigate the decision-making behavior of individuals within an intertemporal task involving loss scenarios. This investigation was conducted under both time pressure and no time pressure condition. Our analysis yielded several significant findings. Firstly, participants exhibited shorter decision-making times under time pressure compared to no time pressure. Additionally, there was a significant decrease in the frequency of choosing the LL option under time pressure in contrast to conditions without time pressure. Moreover, significant differences in eye-tracking patterns were evident between the two experimental conditions, with participants displaying reduced Mean Fixation Duration (MFD) and Search Measure (SM) values when operating under time pressure. Notably, the presence or absence of time pressure appeared to influence the selection of the LL option, with variations in MFD and SM values serving as potential mediators in this relationship.

Time pressure reduces the frequency of choosing the LL option, thus confirming Hypothesis 1 and revealing the asymmetry of losses and gains under time pressure. This outcome may be attributed to several factors. Firstly, time pressure amplifies aversion to losses, intensifying the distress associated with greater losses, thereby reducing the likelihood of choosing a greater loss. Participants’ post-experimental reports of decision-making strategies suggest that individuals may adhere to principles of loss minimization or early loss reduction when making quick decisions. Secondly, under time pressure, individuals may adopt a relatively intuitive, emotional, fast, and effortless mode of information processing that may ignore holistic aspects and focus more on negative information about key dimensions (Svenson and Edland, 1987; Rand, 2016). Other time pressure experiments have found similar effects. Individuals may exhibit more selfish behavior under time pressure (Capraro and Cococcioni, 2016; Krawczyk and Sylwestrzak, 2018; Teoh et al., 2020) or become more pro-social (Rand et al., 2012; Rand, 2016; Bouwmeester et al., 2017).

Under time pressure conditions, individuals exhibit significantly shorter Mean Fixation Duration (MFD) compared to conditions without time pressure, thus confirming Hypothesis 2. MFD represents the average duration of single fixations during decision-making, reflecting the level of cognitive effort and depth of information processing (Velichkovsky, 1999; Horstmann et al., 2009; Amblee et al., 2017). The discrepancy in reaction times also suggests a significant difference in cognitive effort between the two conditions. Meanwhile, participants’ perceived stress scores under the time pressure task averaged 4, which also led to reduced information processing depth. Additionally, prior research has found similar conclusions that individuals spend less cognitive effort when making quick decisions, resulting in relatively shorter reaction times (Sutter et al., 2003; Cappelletti et al., 2011). Conversely, deliberate decision-making strategies typically entail higher cognitive effort, leading to relatively longer reaction times (Horstmann et al., 2009; Su et al., 2013; Zhou et al., 2021). Therefore, the depth of information processing becomes more fragile with increasing time pressure, representing a key manifestation of heuristic strategies.

Under conditions of time pressure, individuals’ Search Measure (SM) significantly increases compared to conditions without time pressure, thereby confirming Hypothesis 3. SM intuitively reflects the direction of information search (Böckenholt and Hynan, 1994) and is frequently utilized in eye-tracking studies to evaluate the general direction of information acquisition (Su et al., 2013; Liu et al., 2021; Zhou et al., 2022). Our finding confirms that under time pressure, individuals adjust their information processing approach, relying more on non-compensatory strategies to adapt to time constraints (Svenson et al., 1990). Similar outcomes have been observed in other studies, such as in consumer brand decision-making, where time pressure prompts individuals to adopt more of a scanning strategy between brands (Pieters and Warlop, 1999). Heuristic-based models generally suggest that as cognitive demands on decision-makers increase (such as under time pressure), decision-makers increasingly rely on heuristic methods based on limited information search (Payne et al., 1996; Rieskamp and Hoffrage, 2008). Consequently, the degree of dimensional processing becomes more pronounced with increasing time pressure, representing a key manifestation of heuristic strategies.

The results of the mediation analysis showed that Mean Fixation Duration (MFD) and Search Measure (SM) served as parallel mediators between task type and the proportion of choosing the LL option. The mediating role of MFD was found to be more pronounced than that of SM, supporting Hypothesis 4. These findings highlight the significance of MFD and SM in decision-making processes influenced by task type. Although MFD and SM do not directly affect decision outcomes, their inclusion in the model remains valuable. They offer insights into cognitive processes and decision-making strategies that are otherwise difficult to observe and measure. Additionally, MFD and SM act as indicators of heuristic decision-making strategies, and their mediation effects suggest that individuals are more likely to adopt heuristic strategies under time pressure. Specifically, time pressure drives rapid information search and superficial information processing, leading to a less frequent selection of the LL option. This can be attributed to the swift comparison of loss magnitudes (SM), alongside reduced reliance on complex computational strategies and cognitive effort (MFD). However, it is important to acknowledge that while MFD and SM offer valuable insights, they do not fully capture the entire cognitive process under time pressure. They provide a glimpse into how time pressure influences decision-making through heuristic strategies, as observed through eye-tracking. Future research should aim to incorporate more comprehensive measures and sophisticated models to deepen our understanding of these cognitive processes.

## 5 Limitations

In terms of limitations, several shortcomings are evident in this study. For instance, while there was a decrease in the proportion of individuals selecting the LL option, the effect size was small. This could be attributed to the randomization of options, potentially compromising the robustness of the effect. Nonetheless, this does not detract from the validity of the main cognitive processes investigated in the study. Moreover, future research should explore whether temporal settings represent a significant research question.

## 6 Conclusion

In conclusion, time pressure significantly influenced decision-making behavior, leading to faster decisions and decreased LL option selection frequency. Eye-tracking data indicated altered information processing strategies under time pressure. The mediation analyses underscored the role of MFD and SM in influencing decision outcomes, highlighting a propensity for heuristic decision-making under time pressure.

## Data Availability

The datasets presented in this study can be found in online repositories. The names of the repository/repositories and accession number(s) can be found in the article/Supplementary material.

## Footnotes

^1^https://osf.io/u7rwv/
